# CTD-dependent and -independent mechanisms govern co-transcriptional capping of Pol II transcripts

**DOI:** 10.1038/s41467-018-05923-w

**Published:** 2018-08-23

**Authors:** Melvin Noe Gonzalez, Shigeo Sato, Chieri Tomomori-Sato, Joan W. Conaway, Ronald C. Conaway

**Affiliations:** 1 Stowers Institute for Medical Research, 1000 E 50 th Street, Kansas City, MO 64110 USA; 2 Department of Biochemistry and Molecular Biology, Kansas University Medical Center, Kansas City, KS 66160 USA

## Abstract

Co-transcriptional capping of RNA polymerase II (Pol II) transcripts by capping enzyme proceeds orders of magnitude more efficiently than capping of free RNA. Previous studies brought to light a role for the phosphorylated Pol II carboxyl-terminal domain (CTD) in activation of co-transcriptional capping; however, CTD phosphorylation alone could not account for the observed magnitude of activation. Here, we exploit a defined Pol II transcription system that supports both CTD phosphorylation and robust activation of capping to dissect the mechanism of co-transcriptional capping. Taken together, our findings identify a CTD-independent, but Pol II-mediated, mechanism that functions in parallel with CTD-dependent processes to ensure optimal capping, and they support a “tethering” model for the mechanism of activation.

## Abstract (web summary)

The co-transcriptional capping of transcripts synthesized by RNA Pol II is substantially more efficient than capping of free RNA, a process that has been shown to depend on CTD phosphorylation. Here the authors demonstrate that a CTD-independent mechanism functions in parallel with CTD-dependent processes to ensure efficient capping.

## Introduction

Messenger RNA and other transcripts synthesized by RNA polymerase II (Pol II) are distinguished by the presence of a 5′-guanosine cap. The cap is added by the capping enzyme to nascent Pol II transcripts bearing 5′-triphosphate ends, where it aids in subsequent steps of RNA maturation, transport, translation, and other processes^1,2^. A remarkable property of the capping reaction is its selectivity for Pol II transcripts. Despite the presence in cells of abundant Pol I and Pol III transcripts with 5′-triphosphate ends, Pol II transcripts are the primary targets for capping by the capping enzyme^2,3^. Consequently, how this exquisite selectivity is accomplished has been of major interest.

An important clue to the selectivity of capping enzyme came from the discovery that co-transcriptional capping of Pol II transcripts is substantially more efficient than capping of free RNA; indeed, the specific activity of capping enzyme for nascent transcripts emerging from elongating Pol II is several orders of magnitude greater than its specific activity for free RNA^4^. This revelation argued that inherent features of the Pol II transcription complex are responsible for dramatically activating capping and, in so doing, ensuring selectivity of capping enzyme.

Though it is presently not known exactly why co-transcriptional capping is so efficient, previous studies have implicated the phosphorylated Pol II CTD in both recruitment and activation of the capping enzyme^5,6^. Pol II is distinguished from Pol I and Pol III by the presence of a unique carboxyl-terminal domain (CTD) on its largest subunit, RPB1. The Pol II CTD, conserved from yeast to humans, consists of a tandemly repeated heptapeptide motif with consensus sequence Y_1_S_2_P_3_T_4_S_5_P_6_S_7_, which is subject to extensive phosphorylation.

Additional studies have shed considerable light on biochemical mechanisms underlying activation of co-transcriptional capping of Pol II transcripts, leading to the formulation of several non-mutually exclusive models for how the phosphorylated CTD might activate capping. One model proposes that the phosphorylated CTD activates capping by recruiting and tethering the capping enzyme to elongating Pol II in the vicinity of the emerging nascent transcript (tethering model). This model is supported by evidence that the capping enzyme binds specifically and stably to GST-CTD or CTD heptapeptides phosphorylated at either serine 2 (pSer2) or serine 5 (pSer5)^5,7–9^. A second model proposes that the pSer5-CTD activates capping by allosterically activating capping enzyme (allosteric activation model). This model is supported by evidence that binding of capping enzyme to CTD heptapeptide repeats phosphorylated on serine 5, but not on serine 2, increases formation of the covalent capping enzyme–GMP complex, an intermediate during transfer of the 5′-guanosine cap to Pol II transcripts^8,10^.

Despite this evidence, the relative importance of CTD phosphorylation for activation of capping has been questioned, since blocking CTD phosphorylation only partially inhibits co-transcriptional capping. CDK7, the protein kinase associated with the Pol II initiation factor TFIIH, preferentially phosphorylates CTD Ser5 and Ser7^11^. Addition of CDK7 inhibitors to block CTD phosphorylation in transcription complexes assembled in nuclear extracts led to only a modest reduction in capping efficiency^4,12^, suggesting that other activation mechanisms likely contribute to the activation of RNA capping.

In this report, we exploit a defined Pol II transcription system that supports both CTD phosphorylation and robust activation of co-transcriptional capping to dissect the mechanism of capping. As described below, our findings define a CTD-independent mechanism that functions in parallel with CTD-dependent processes to ensure maximal capping. In addition, we report mechanistic experiments that argue that a combination of CTD-independent and CTD-dependent tethering mechanisms likely play a dominant role in activation of co-transcriptional capping.

## Results

### Co-transcriptional capping in a minimal Pol II transcription system

**Fig. 1:**
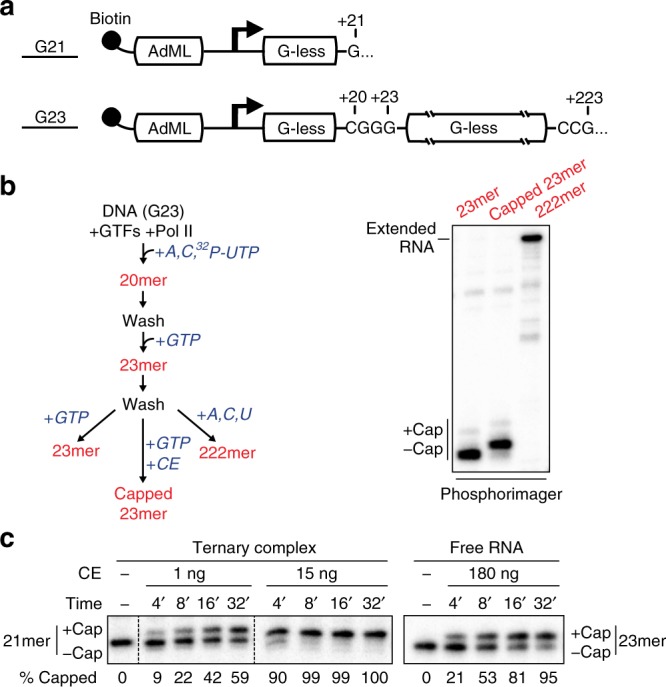
Co-transcriptional capping activation in a defined enzyme system. **a** Biotinylated DNA templates used for promoter-dependent transcription. Both contain the Adenovirus 2 Major Late core promoter (AdML) followed by one (G21) or two (G23) G-less cassettes. **b** 23mer transcripts in washed ternary complexes were prepared according to the diagram and incubated with GTP (lane 1), GTP and 5 ng of capping enzyme (CE) (lane 2), or ATP, CTP, and UTP (lane 3). In this and subsequent figures, radiolabeled transcripts were resolved by denaturing gel electrophoresis and detected using a phosphorimager. **c** Kinetics of co-transcriptional capping and capping of free RNA. Free RNA or washed ternary complexes containing 21mers were incubated for varying lengths of time with 50 µM GTP and the indicated amounts of capping enzyme (CE). % Capped RNA is the quantification of a single representative experiment

To investigate the mechanism underlying activation of co-transcriptional capping of Pol II transcripts, we used a defined Pol II transcription system consisting of purified RNA polymerase II and TFIIH, recombinant TBP, TFIIB, TFIIE, and TFIIF, and DNA templates containing the adenovirus 2 major late promoter (AdML) followed by one (G21) or two (G23) G-less cassettes (Fig. 1a).

Immobilized G23 templates were incubated with Pol II and initiation factors to assemble preinitiation complexes (PICs). ATP, α-^32^P-UTP, and CTP were then added to allow synthesis of 20 nucleotide (nt) transcripts and then washed to remove unincorporated rNTPs. 20mers were walked to 23mers by addition of GTP and washed again. The resulting ternary transcription complexes were incubated with or without recombinant mammalian capping enzyme and with GTP, the GMP donor for capping. Capping of nascent transcripts was monitored by an assay that detects an electrophoretic mobility shift of ~1 nt in capped transcripts^13,14^, indicating addition of a 5′ cap (Fig. 1b, compare first two lanes). Confirming the 23mers used as substrates for RNA capping were associated with transcribing Pol II, they were chased quantitatively into longer transcripts upon addition of ATP, CTP, and UTP to allow transcription to traverse the second G-less cassette in the G23 template (Fig. 1b, third lane). Digestion of these RNA products with cap-sensitive phosphatases and exonucleases confirmed the shift in mobility was due to capping of nascent transcripts (Supplementary Fig. 1). Furthermore, and consistent with previous findings^4^, we observed that the specific activity of capping enzyme for transcripts associated with the Pol II transcription complex is substantially greater than for free RNA. Fifteen nanograms of capping enzyme were sufficient to cap 90% of transcripts in co-transcriptional capping reactions in 4 min, while it took more than 30 min to achieve a similar amount of capping of free RNA with 180 ng of capping enzyme (Fig. 1c).

### TFIIH kinase activates co-transcriptional capping

Because previous studies implicated phosphorylation of the Pol II CTD as a key step in capping activation, we explored the contribution of CTD phosphorylation to capping in our minimal Pol II transcription system, where the TFIIH-associated CDK7 kinase is solely responsible for CTD phosphorylation.

**Fig. 2:**
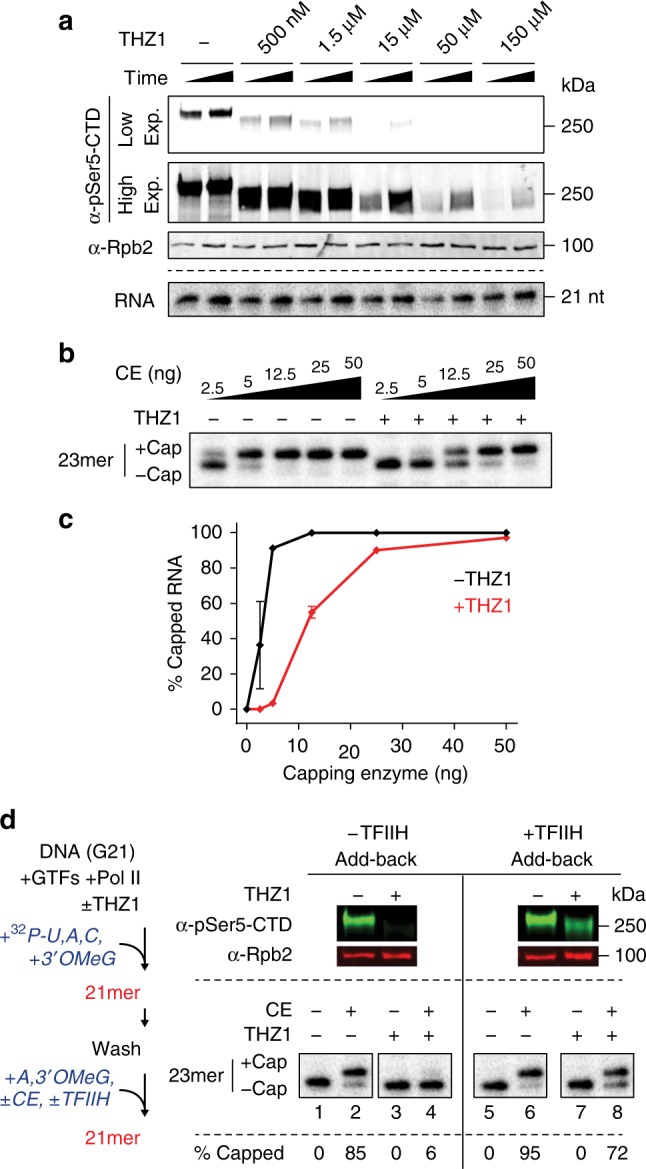
Cdk7 strongly stimulates co-transcriptional capping in the purified enzyme system. **a** 21mers were synthesized in parallel reactions with unlabeled (upper panel) or radiolabeled (lower panel) ribonucleoside triphosphates in the presence of DMSO (−) or increasing amounts of THZ1; reactions were stopped after 15 or 60 min. Upper panel, reaction products were analyzed by western blotting using antibodies against Ser5-phosphorylated Rpb1 (α-pSer5-CTD); two different exposures of the same image are shown. As a control for equal loading of Pol II in each lane, the same blot was probed with antibodies against Rpb2. Lower panel, radiolabeled transcripts were analyzed on denaturing gels and detected by phosphorimaging. **b** Washed transcription complexes containing 23mers synthesized with or without 150 µM THZ1 were incubated for 4 min with GTP and increasing amounts of capping enzyme (CE). **c** Graph shows mean and range of two independent reactions performed as in **b**. **d** As diagrammed on the left, transcription complexes containing 21mers were prepared in the presence of DMSO or 100–150 µM THZ1, washed, and incubated for 15 min with 50 µM ATP and 100 µM 3′OMeG, with or without 3 ng of capping enzyme. For +TFIIH add-back reactions (lanes 5–8), 300 ng of purified TFIIH was added with capping enzyme. Reactions were assayed for Pol II CTD phosphorylation status by western blotting (top) or for RNA capping (bottom). % Capped indicates average of two independent reactions

THZ1 is a covalent inhibitor of CDK7 and of CDK7-dependent serine 5 phosphorylation on the Rpb1 CTD in vitro and in vivo^15^. To begin to investigate the contribution of CTD phosphorylation in our defined enzyme system, preinitiation complexes were assembled on immobilized templates, and G-less transcripts were synthesized with or without THZ1. Addition of THZ1 decreased Ser5 phosphorylation on the CTD (pSer5-CTD) during transcription by ~50% at 500 nM, by ~90% at 1.5 µM, and achieved near-complete inhibition by 150 µM (Fig. 2a). Similar results were obtained when CTD phosphorylation was assayed with γ-^32^P-ATP as phosphate donor, indicating that the vast majority of CTD phosphorylation is inhibited by THZ1 in these assays (Supplementary Fig. 2A). As expected from previous results demonstrating that CTD phosphorylation is not required for basal transcription with purified factors in vitro^16^, THZ1 had no major effect on RNA synthesis even at the highest concentration used (Fig. 2a).

Addition of THZ1 substantially decreased, but did not completely inhibit, co-transcriptional capping in the reconstituted enzyme system. Without THZ1, 5 ng of capping enzyme was sufficient to cap nearly all transcripts, while ~5 times more capping enzyme was needed to achieve a similar level of capping with 150 µM THZ1 (Fig. 2b, c). We used 150 µM THZ1 in this and subsequent assays because residual CTD phosphorylation remained in reactions with less inhibitor; however, capping was substantially inhibited at THZ1 concentrations as low as 1 µM (Supplementary Fig. 2B). To control for off-target effects of THZ1, we included it in many control reactions during all steps after pulse labeling and CTD phosphorylation. Notably, even in the presence of THZ1, co-transcriptional capping was still substantially more efficient than capping of free RNA.

To ensure that the TFIIH kinase is the target of THZ1-dependent inhibition of capping, we performed a TFIIH add-back experiment. Transcription complexes containing 21mers were synthesized in the presence or absence of THZ1 as diagrammed in Fig. 2d. After washing to remove THZ1, capping reactions were performed with or without addition of new TFIIH and in the presence of a low concentration of capping enzyme (5 ng). As expected, THZ1 treatment inhibited Ser5-CTD phosphorylation (Fig. 2d, upper panel) and RNA capping (Fig. 2d, lower panel). Add-back of untreated TFIIH, following THZ1 inhibition, rescued Ser5-CTD phosphorylation and RNA capping.

### TFIIH kinase activates capping in artificial ternary complexes

Thus far our results indicate that the TFIIH kinase can activate co-transcriptional capping of transcripts initiated by Pol II from a promoter in the presence of a minimal set of initiation factors. A limitation of these assays is that TFIIH is required not only for CTD phosphorylation, but also for transcription initiation; moreover, using these assays we cannot distinguish between the possibilities that (i) the residual TFIIH kinase activity observed even in the presence of high THZ1 concentrations is sufficient to activate co-transcriptional capping or (ii) phosphorylation-dependent and -independent events activate co-transcriptional capping.

We therefore sought to simplify the transcription system further using artificial Pol II ternary elongation complexes pre-assembled on synthetic DNA:RNA transcription bubbles, which allow transcription without a promoter and without general transcription factors. This methodology has been successfully used to study structures and function of ternary complexes assembled with budding yeast and mammalian Pol II (e.g., refs. ^17–20^) and to obtain an EM structure of fission yeast Pol II bound to capping enzyme^21^. As discussed below, we found that activation of co-transcriptional capping of transcripts associated with artificial ternary complexes was as robust as activation in the minimal Pol II transcription system.

**Fig. 3:**
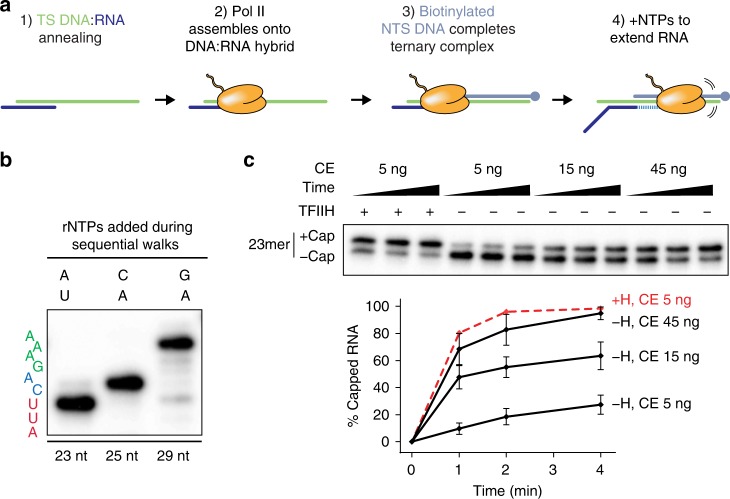
TFIIH-dependent activation of co-transcriptional capping in artificial ternary complexes assembled on DNA:RNA scaffolds. **a** Scheme for assembly of artificial ternary elongation complexes on DNA:RNA scaffolds. See text and “Methods” for details. **b** Representative gel image showing step-wise increase of RNA length following addition of appropriate combinations of rNTPs. 20-nt synthetic RNA in artificial ternary complexes was extended to position +23 with 5 µM ATP and 10 µCi α-^32^P-UTP (first lane), washed and walked to position +25 with 20 µM CTP and ATP (second lane), and finally washed and walked to position +29 with 20 µM ATP and GTP (last lane). Sequence on left denotes RNA sequence from +21 (bottom) to +29 (top). **c** 23mers in artificial ternary complexes were incubated with buffer or 300 ng of purified TFIIH for 10 min, washed, and incubated for 1, 2, or 4 min with 50 µM GTP and the indicated amounts of capping enzyme. Graph shows mean and range of data from two independent reactions

DNA template strand and 20 nt RNA oligonucleotides with 5′-triphosphate ends were annealed to form DNA:RNA hybrids, incubated with purified mammalian Pol II to position the enzyme on the duplex, and supplemented with a molar excess of biotinylated DNA non-template strand to close the ternary complex (Fig. 3a). Immobilized ternary complexes were then washed to remove unbound Pol II, DNA, and RNA, followed by addition of rNTPs to walk the RNA to the desired length (Fig. 3b).

Co-transcriptional capping in ternary complexes assembled on DNA:RNA transcription bubbles recapitulated features of capping of promoter-specific transcripts in our defined enzyme system. Although TFIIH was dispensable for co-transcriptional capping in these ternary complexes, including it increased the specific activity of capping enzyme by approximately tenfold (Fig. 3c and Supplementary Fig. 3). Moreover, THZ1 blocked the vast majority of CTD phosphorylation (Supplementary Fig. 4A) and reduced co-transcriptional capping in reactions containing TFIIH (Supplementary Fig. 4B), indicating that CDK7 catalytic activity is needed for TFIIH-dependent activation of capping in ternary complexes. Thus, TFIIH-dependent activation of capping does not require initiation from a promoter and is independent of other initiation factors.

### Direct evidence the Pol II CTD is the target of the TFIIH kinase

Our finding that Pol II ternary complexes assembled on DNA:RNA transcription bubbles faithfully recapitulate co-transcriptional capping in the absence of a promoter and general transcription factors argues that (i) TFIIH-dependent capping activation depends solely on features of the Pol II elongation complex and (ii) Pol II is the sole target of protein kinase activity required for capping activation.

**Fig. 4:**
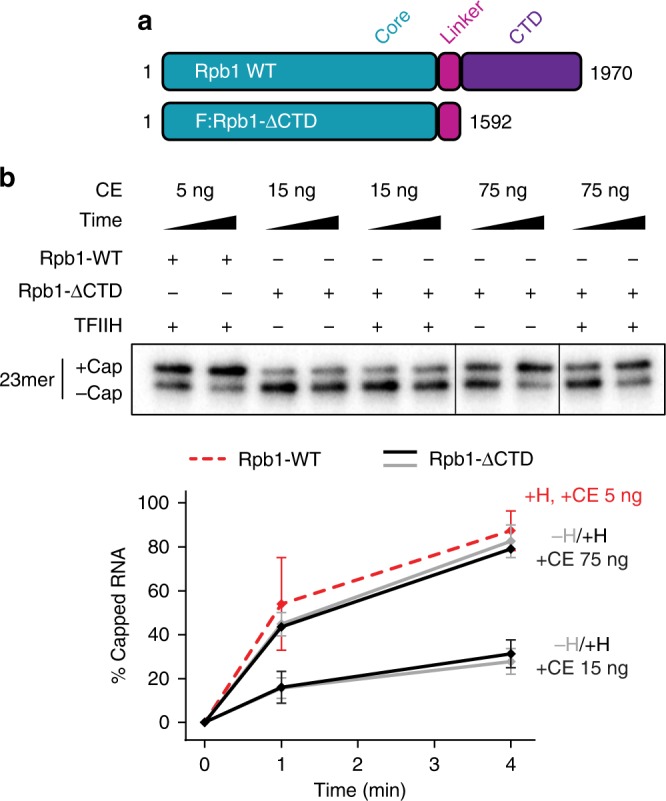
Pol II CTD is necessary for TFIIH-dependent activation of co-transcriptional capping. **a** Diagram of wild-type (WT) Rpb1 and FLAG-tagged Rpb1 mutant lacking the CTD (F:Rpb1-ΔCTD). Numbers denote amino acid positions. **b** Artificial ternary complexes containing 23mers were assembled with WT RNA Pol II (Rpb1-WT) or RNA Pol II lacking the CTD (Rpb1-ΔCTD), incubated with buffer or 300 ng of purified TFIIH for 10 min, washed, and then incubated for 1 or 4 min with 50 µM GTP and the indicated amounts of capping enzyme. Of total 30 µl reaction, 5 µl and 30 µl were loaded for WT and ΔCTD Pol II, respectively. All lanes are from the same gel; for consistency of presentation, the seventh and eighth lanes (Rpb1ΔCTD, 75 ng capping enzyme) are shown as a mirror image of their orientation in the original gel image. Graph shows mean and range of two independent reactions

To test directly whether the Pol II CTD is the target of the TFIIH kinase, we assayed capping in ternary complexes assembled with mutant Pol II lacking the CTD. To accomplish this, we prepared CTD-deficient Pol II from a cell line expressing a FLAG-tagged Rpb1 mutant lacking the entire CTD (Fig. 4a and Supplementary Fig. 5).

Without TFIIH, capping was similarly efficient in ternary complexes containing Pol II with or without the CTD (compare Figs. 3c and 4b). Whereas TFIIH strongly stimulated capping in ternary complexes containing wild-type Pol II, it had no effect on either the rate or extent of capping when added to ternary complexes assembled with CTD-deficient Pol II (Fig. 4b), indicating that the CTD is the target for TFIIH-dependent capping activation. Nevertheless, co-transcriptional capping in the absence of TFIIH and the CTD (Fig. 4b) remains much more efficient than capping of free RNA (Fig. 1c), arguing that additional features of the ternary complex, independent of the CTD, also contribute to capping activation. This observation is consistent with a prior report that capping in transcription complexes that had been assembled in nuclear extracts, washed with high salt, and treated with chymotrypsin to remove the CTD is more efficient than capping of free RNA^4^. These prior studies did not, however, rule out the possibilities that (i) factor(s) other than the ternary complex remain after high salt washes and enhance capping or (ii) one or a few CTD repeats remain after proteolysis.

### Species-specific interactions support co-transcriptional capping

To explore the nature of CTD-independent capping activation, we considered the possibility that proper presentation of the 5′-triphosphate ends of transcripts emerging from the Pol II exit channel is important for CTD-independent capping activation. Alternatively, capping enzyme could have binding sites on Pol II other than the phosphorylated CTD. Indeed, structural studies have provided evidence for contacts between yeast capping enzyme and surfaces on the body of yeast Pol II, either in the multihelical foot domain of Rpb1^22^ or near the RNA exit channel^21^. Such contacts might enhance capping by positioning the capping enzyme so that it can capture the 5′-end of the nascent transcript as it emerges from the RNA exit channel; however, the contribution of Pol II body-capping enzyme interactions to co-transcriptional capping has not been explored.

If phospho-CTD-independent capping activation depends solely on the conformation of the nascent transcript as it emerges from the Pol II exit channel, one would expect that capping by mammalian capping enzyme would be insensitive to the source of Pol II used to assemble ternary complexes. In contrast, if co-transcriptional capping depends on contacts between capping enzyme and surfaces in the Pol II body, maximal CTD-independent capping by mammalian capping enzyme might be achieved only with elongation complexes containing its cognate Pol II.

**Fig. 5:**
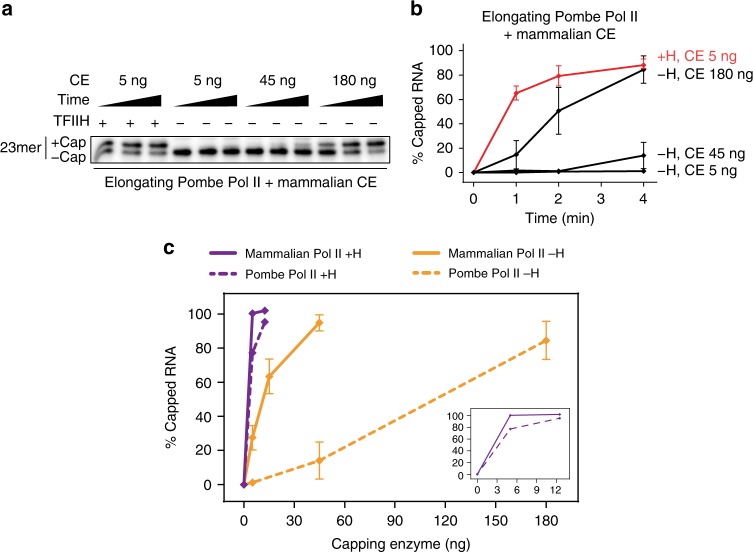
Species-specific interactions between mammalian capping enzyme and artificial ternary complexes assembled with *S. pombe* Pol II. **a** Artificial ternary complexes were assembled with *S. pombe* RNA Pol II, following the same protocol used for mammalian Pol II. Washed complexes containing 23mers were then incubated with buffer or 300 ng of purified TFIIH for 10 min, washed, and incubated with 50 µM GTP and capping enzyme for 1, 2, or 4 min. **b** Graph shows mean and range of 2 independent reactions performed as in **a**. **c** Artificial ternary complexes containing mammalian Pol II (solid lines) or *S. pombe* Pol II (dotted lines) were incubated for 4 min with various concentrations of capping enzyme, with (purple) or without (orange) TFIIH. Graph shows mean and range from two independent reactions. Inset shows only reactions with TFIIH

To address these possibilities, we assayed capping by mammalian capping enzyme using artificial elongation complexes assembled with either mammalian Pol II or Pol II from the evolutionarily distant fission yeast *Schizosaccharomyces pombe*. Without TFIIH-dependent CTD phosphorylation, capping in ternary complexes containing fission yeast Pol II was reduced to a much greater extent than in complexes containing mammalian Pol II: ~10 times more capping enzyme was needed to cap 50% of transcripts in fission yeast ternary complexes than to cap the same fraction of transcripts in mammalian ternary complexes (Fig. 5c, compare orange lines at 50%). Notably, under conditions that support complete capping of transcripts in mammalian Pol II ternary complexes (Fig. 3c, 4 min reactions, ~45 ng of capping enzyme), there was almost no capping of transcripts in ternary complexes with fission yeast Pol II (Fig. 5a, b). While it is formally possible that these differences in capping efficiency in the absence of TFIIH could be explained at least in part by differences in the number of CTD repeats in Pol II from *S. pombe* (29 repeats) and rat (52 repeats), we think this is unlikely because interactions between capping enzyme and CTD repeat-containing peptides or proteins have been shown to depend on CTD phosphorylation^5–10^. Instead, we believe our findings suggest that contacts between capping enzyme and the body of Pol II contribute to co-transcriptional capping.

Interestingly, when reactions were carried out in the presence of TFIIH to phosphorylate the Pol II CTD, capping was similarly efficient in ternary complexes containing either mammalian or fission yeast Pol II (Fig. 5c, purple lines). That TFIIH-dependent phosphorylation of the *S. pombe* Pol II CTD can restore capping activation by mammalian capping enzyme to levels seen with mammalian Pol II suggests crosstalk between phospho-CTD-dependent and -independent mechanisms. Together, our findings are consistent with the model that evolutionarily conserved interaction of capping enzyme with the Pol II CTD, as well as species-specific interaction of capping enzyme with Pol II surface(s) outside the phospho-CTD, contribute to capping activation.

### A tethering model can account for activation of capping

Thus far our results argue that both CTD-dependent and CTD-independent mechanisms activate co-transcriptional capping. Our results are consistent with the possibility that interaction(s) of capping enzyme with Pol II surfaces on the phospho-CTD and elsewhere are the major determinants of capping activation, but they shed no light on how these interactions activate capping.

To address this question, we performed a series of experiments to explore two current, non-mutually exclusive activation models, which we refer to as the tethering and allosteric activation models. The tethering model argues that Pol II elongation complexes act as scaffolds to bring capping enzyme and nascent transcript 5′-triphosphate ends into close proximity, effectively increasing the local concentrations of capping enzyme and transcript. This model does not require that the intrinsic catalytic activity of capping enzyme must be increased to account for capping activation. The allosteric activation model argues that interaction of capping enzyme with site(s) on Pol II is required to increase capping enzyme’s specific activity.

A distinction between these activation models is their prediction for the fate of transcripts added in *trans*. The tethering model requires that both capping enzyme and nascent transcript be bound to the same Pol II scaffold for capping activation to occur. Thus, free RNA added in *trans* to ternary complexes would be capped as inefficiently as free RNA alone. In contrast, the allosteric activation model requires simply that capping enzyme be bound to site(s) on Pol II to increase its intrinsic catalytic activity and predicts that it should be possible to activate capping in *trans*.

**Fig. 6:**
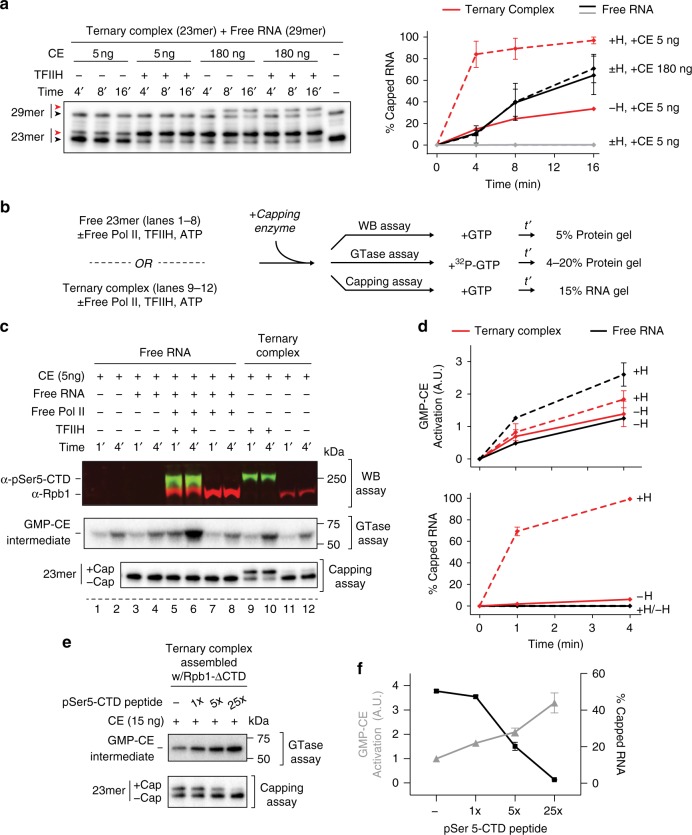
Phosphorylated Pol II provided in *trans* does not activate capping of free RNA. **a** Kinetics of capping of free 29mer RNA and 23mers in artificial ternary complexes, in the same reactions. Ternary complexes containing 23mers were incubated with or without 300 ng of purified TFIIH, washed, and resuspended in buffer containing 50 µM GTP. 29mer RNA was added in *trans* to these reactions, mixed, and reaction mixtures were transferred to new tubes containing capping enzyme. Red and black arrowheads represent capped and uncapped RNA, respectively. Graph shows mean and range of data from two independent reactions. **b** Protocol used in assays shown in **c** and **d**. Capping enzyme was mixed with buffer; with free 23mer RNA, in the presence or absence of pre-phosphorylated Pol II; or with 23mer RNA in phosphorylated artificial ternary complexes. Reactions were supplemented with 50 µM unlabeled GTP to assay CTD phosphorylation (WB assay) and capping or 0.3 µM α-^32^P-GTP to assay guanylyltransferase activity (GTase). *t’* time. **c** Analysis of the reactions prepared in **b** by western blotting using antibodies against Rpb1 (α-Rpb1) or against Ser5-phosphorylated Rpb1 CTD (α-pSer5-CTD), formation of α-^32^P GMP-capping enzyme intermediate, or RNA capping. **d** Quantification of GMP-capping enzyme intermediate formation (top) or total RNA capped (bottom) from **c**. Graphs show mean and range of two independent replicas of assays shown in lanes 5–12. **e** Parallel GTase and capping assays were performed on artificial ternary complexes assembled using Pol II lacking the CTD (Rpb1-ΔCTD). Assays were supplemented with pSer5-CTD peptide or buffer and contained a total of 50 µM GTP. 1× = ~200 ng peptide. **f** Quantitation of reactions performed as in **e**. Graph shows mean and range of two independent replicas

We asked whether free RNA added in *trans* to Pol II elongation complexes was capped with similar efficiency as transcripts associated with these complexes. First, we estimated the relative specific activities of capping enzyme for free RNA alone or free RNA mixed in *trans* with artificial ternary complexes. At saturating concentrations of capping enzyme (180 and 540 ng capping enzyme), free RNA capping required reaction times ~4–8 times longer than needed for similar capping in ternary complexes with just 5 ng capping enzyme (Supplementary Fig. 6). In Fig. 6a, free 29 nt RNA was added in *trans* to ternary complexes containing 23 nt transcripts. As expected, TFIIH strongly activated co-transcriptional capping of 23mers associated with Pol II elongation complexes. In contrast, free 29mers added to ternary complexes in *trans* were capped as inefficiently as free RNA alone, with or without TFIIH. Thus, the specific activity of capping enzyme for free RNA was unaffected by the presence of either TFIIH or a phosphorylated Pol II elongation complex.

Evidence for the allosteric activation model comes from the finding that binding of capping enzyme to phospho-CTD heptapeptide repeats increases formation of covalent GMP-capping enzyme intermediates^8,10^. Mammalian capping enzyme is a bifunctional enzyme possessing both RNA 5′-triphosphatase and guanylyltransferase (GTase) activities. In the first step of capping, the triphosphatase hydrolyzes the RNA 5′-triphosphate to produce a diphosphate. GTP is then loaded into the GTase catalytic center and hydrolyzed to GMP, forming a GMP-capping enzyme intermediate. Finally, GTase transfers GMP to the 5′-diphosphate end of the RNA to form the cap^2,23^.

To assess the correlation between allosteric activation of GTase and capping, we performed capping reactions and measured reaction products using assays that quantified Pol II phosphorylation, GMP-capping enzyme intermediate, or RNA capping (Fig. 6b). We observed a modest increase in formation of the GMP-capping enzyme intermediate in the presence of Pol II with Ser5-phosphorylated CTD; however, this increase did not correlate with an increase in the efficiency of free RNA capping (Fig. 6c, d). In particular, addition of CTD-phosphorylated, but not unphosphorylated, Pol II in *trans* led to an approximately twofold increase in GMP-capping enzyme intermediate, although there was no detectable capping of free RNA. Furthermore, GMP-capping enzyme intermediate formation was increased less than twofold by CTD phosphorylation in reactions containing Pol II ternary complexes, while co-transcriptional capping was greatly enhanced.

As a second test of the correlation between allosteric activation of GTase and capping, we asked whether capping of nascent transcripts associated with elongating, CTD-less Pol II was enhanced in the presence of a CTD peptide consisting of five Ser5-phosphorylated heptamer repeats (Fig. 6e, f). We incubated ternary complexes containing CTD-less Pol II with capping enzyme and varying concentrations of Ser5-phosphorylated peptide and, in parallel reactions, measured formation of GMP-capping enzyme intermediate or capped RNAs. Addition of Ser5-phosphorylated CTD peptide increased GMP-capping intermediate formation approximately two–fourfold, depending on peptide concentration. We anticipated this increase would have no effect on capping of nascent transcripts in CTD-less ternary complexes or, if allostery makes a major contribution to capping activation, would enhance the rate of capping. Surprisingly, however, we observed that the peptide inhibited capping at concentrations that led to the largest increase in GMP-capping enzyme intermediate.

The results presented thus far indicate that (i) free RNA capping is not stimulated by free phosphorylated Pol II or by active ternary complexes provided in *trans* and (ii) changes in rates of formation of GMP-capping enzyme when phospho-CTD is provided in *trans* do not correlate with changes in the rate of capping of either free RNA or RNA in ternary complexes. Together, these observations suggest that allosteric activation of capping enzyme through interaction with phospho-CTD plays a relatively minor role in co-transcriptional capping activation in our system.

The tethering model suggests that proximity of the RNA 5′-end to the Pol II elongation complex might contribute to capping activation. If so, one might expect capping of long transcripts, whose 5′-ends have moved away from the RNA exit channel, would be less efficient than capping of short transcripts.

**Fig. 7:**
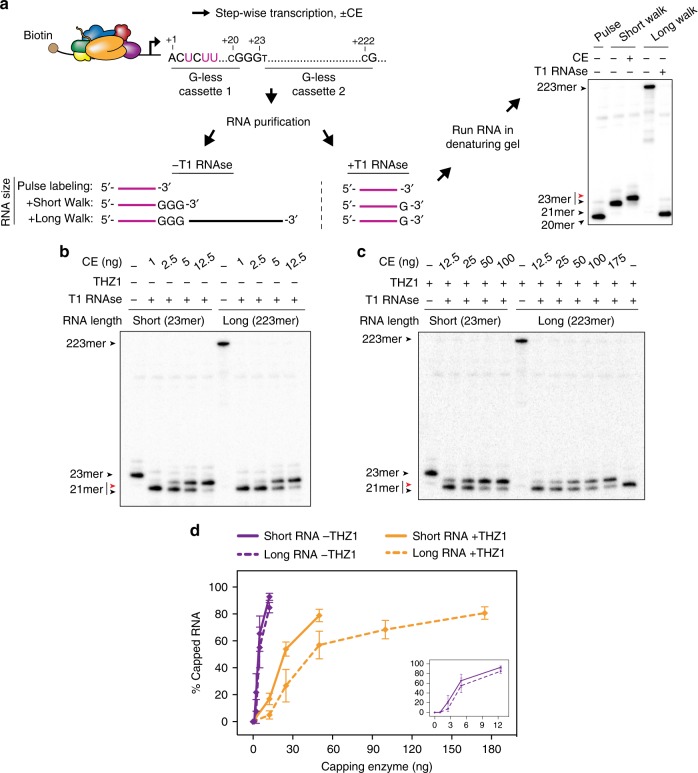
RNA-length dependent co-transcriptional capping. **a** Diagram of method used to assay co-transcriptional capping of short and long RNAs. Transcripts initiated at the AdML promoter on the G23 DNA template were radiolabeled during an initial pulse labeling step and extended to 23mers or 222mers to generate ternary complexes containing short or long RNAs, respectively. After incubation with buffer or capping enzyme, short (23-nt) and long RNAs (223-nt) were purified and treated for 15 min with T1 RNAse before denaturing gel electrophoresis. Image is from the same gel as that shown in Fig. 1b. Purple lines and “U” represent radiolabeled RNAs and nucleotides. Here and in **b** and **c** red and black arrowheads represent capped and uncapped RNA, respectively. **b**, **c** Ternary complexes containing short (23mer) or long (223mer) RNAs were incubated with 50 µM GTP and varying amounts of capping enzyme for 2 min, and reaction products were processed as in **a**. For the reactions shown in **c**, THZ1 was included in all buffers except during washing. **d** The graph shows mean (±S.D.) from three (reactions without THZ1) or five (with THZ1) independent experiments performed as in **b** and **c**. Solid lines, capping of 23mers; dotted lines, capping of 223mers, with (orange) or without (purple) THZ1. Inset shows only reactions without THZ1

Because we found it technically challenging to generate ternary complexes containing long RNA transcripts using DNA:RNA transcription bubbles, we used our reconstituted enzyme system to compare co-transcriptional capping of short and long transcripts initiated from the promoter on the G23 template, which contains two sequential G-less cassettes. As outlined in Fig. 7a, we synthesized radiolabeled transcripts of 20 nt and washed the resulting transcription complexes to remove initiation factors and excess nucleotides. Transcripts were extended with unlabeled NTPs to 23mers (short walk) or 223mers (long walk), and ternary complexes were incubated with capping enzyme. Since the difference between electrophoretic mobilities of long capped and uncapped transcripts was too small to measure, we included an enzymatic cleavage step post capping. Reaction products were digested with ribonuclease T1, which cleaves after G residues and shortens both 23mers and 223mers to 21 nt, allowing us to detect and quantify capping of both short and long transcripts.

Using this approach, we compared the efficiencies of capping of short and long RNAs, with and without CTD kinase inhibitor THZ1 (Fig. 7b, c). As shown in Fig. 7d, the efficiency of capping of short and long RNA was indistinguishable without THZ1, under conditions of maximal CTD phosphorylation. However, in the presence of THZ1 levels that inhibit CTD phosphorylation more than 95%, increasing the length of nascent RNA reduced the efficiency of capping, although not as dramatically as substituting *S. pombe* Pol II for mammalian Pol II (approximately two–threefold vs approximately tenfold).

The results presented thus far are consistent with the model that the Pol II elongation complex acts as a scaffold that brings capping enzyme and the 5′-end of the nascent transcript together. However, it is formally possible that our observation that capping of free RNA or RNA in CTD-less Pol II elongation complexes cannot be activated in *trans* is due not to a requirement that the nascent transcript be tethered to the Pol II elongation complex, but rather that passage of the nascent transcript 5′-end through the RNA exit channel of wild-type Pol II during transcript synthesis allows it to adopt a conformation needed for optimal capping. To address this possibility, we investigated the effect on capping of using ribonuclease T1 cleavage to untether the nascent transcript prior to capping.

**Fig. 8:**
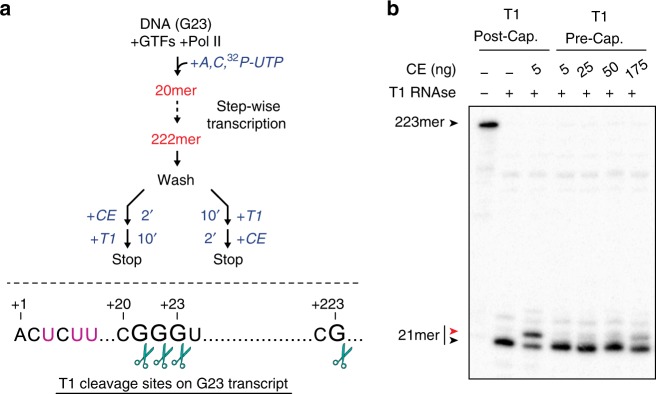
Release of nascent RNA from ternary complex prevents capping activation. **a** (Top) Diagram of reaction scheme. Ternary complexes initiated from the AdML promoter were walked to position +222, washed, resuspended in buffer with 50 µM GTP, and treated with T1 RNase for 10 min post capping or pre-capping. When T1 was added after capping, reactions were incubated with 5 ng of capping enzyme for 2 min, then incubated with T1 RNase and 10 mM EDTA for 10 min. When T1 was added before capping, reactions were incubated with T1 RNase for 10 min, then incubated with the indicated amounts of capping enzyme for 2 min. (Bottom) Diagram of the RNA sequence associated with ternary complexes, showing T1 cleavage sites (scissors). Purple indicates radiolabeled nucleotides. **b** Analysis of the capping reactions prepared in **a**. Red and black arrowheads represent capped and uncapped RNA, respectively

As diagrammed in Fig. 8a, we generated wild-type Pol II elongation complexes with long RNA (Fig. 8b, lane 1). Complexes were washed and incubated with T1 before capping to generate shorter RNA fragments that have passed through the RNA exit channel during synthesis but are untethered from the elongation complex, or, in control reactions, after capping. As expected, 5 ng of capping enzyme was sufficient to cap ~50% of transcripts tethered to ternary complexes (Fig. 8b, compare second and third lanes). When nascent transcripts were untethered from elongation complexes by treatment with T1 before addition of capping enzyme, capping efficiency was dramatically reduced even at higher concentrations of capping enzyme (Fig. 8b, last lane). Thus, untethering nascent RNA from Pol II elongation complex was sufficient to render the efficiency of capping similar to that observed with free RNA, providing further support for the tethering model for activation of co-transcriptional capping.

## Discussion

Here, we investigated the biochemical mechanisms underlying co-transcriptional capping activation of mammalian Pol II transcripts. Our findings support the model that activation of co-transcriptional capping is primarily due to tethering the nascent transcript and capping enzyme to transcribing Pol II via contacts with both Ser5-phosphorylated CTD and yet-to-be-defined sites on the body of Pol II.

First, we observe robust capping activation of nascent transcripts in reactions containing purified Pol II assembled into artificial ternary complexes. Co-transcriptional capping of transcripts in these artificial ternary complexes is enhanced about tenfold in the presence of catalytically active TFIIH CDK7 kinase. We note that blocking CDK7 activity with THZ1 in promoter-dependent assays leads to an approximately fivefold decrease in capping efficiency. This difference is likely due to residual CTD kinase activity in reactions containing THZ1, since as shown in Fig. 2a THZ1 greatly reduces, but does not completely inhibit, CTD phosphorylation. TFIIH-dependent capping activation is not observed in reactions containing mutant Pol II lacking CTD, providing strong support for the notion that the Pol II CTD is the sole target for phosphorylation-dependent activation of co-transcriptional capping in this system.

Second, our observation that the Pol II CTD contributes to capping activation only in the presence of TFIIH and when CDK7 phosphorylates the CTD is consistent with previous studies arguing that the phosphorylated CTD plays an important role in activation of co-transcriptional capping^5–10^. Consistent with these early observations, a recent study demonstrated that CTD Ser5 phosphorylation and capping enzyme occupancy at the 5′-end of genes is reduced genome-wide in human cells expressing an analog-sensitive CDK7 mutant^24^. These studies brought to light two potential roles for the phosphorylated CTD in activation of capping. First, capping enzyme is recruited to the Pol II elongation complex via specific and stable binding to the phosphorylated CTD. In addition, capping enzyme can be allosterically activated to form the capping enzyme–GMP intermediate upon interaction with Ser5-phosphorylated CTD peptides. Based on our evidence that (i) addition in *trans* of Pol II elongation complexes, with or without TFIIH, had no effect on efficiency of free RNA capping, (ii) ribonucleolytic release of nascent transcripts from Pol II elongation complexes abolished capping activation, and (iii) CTD phosphorylation has a much greater effect on capping rate than on capping enzyme–GMP intermediate formation, the critical role of the phospho-CTD in activation of co-transcriptional capping is most likely recruitment and tethering of capping enzyme to Pol II. Nevertheless, it remains possible that allosteric activation of capping enzyme upon its interaction with phospho-CTD in the ternary complex makes a modest contribution to activation of co-transcriptional capping.

Third, robust activation of co-transcriptional capping occurs even in the absence of Pol II CTD or TFIIH, supporting the model that there is a parallel CTD-independent mechanism for activation of capping. That Pol II from the fission yeast *S. pombe* fails to support robust activation of co-transcriptional capping by mammalian capping enzyme unless the CTD is phosphorylated is consistent with the ideas that (i) species-specific interactions of mammalian capping enzyme with mammalian Pol II are critical for this CTD-independent mechanism for activation of capping and (ii) tethering of capping enzyme to Pol II is governed by a multipartite Pol II-binding site, which includes the phosphorylated CTD and a site(s) outside the CTD. Notably, phosphorylation of the fission yeast Pol II CTD is sufficient to restore co-transcriptional capping activation by mammalian capping enzyme to levels seen with mammalian Pol II, indicating that the CTD-dependent and CTD-independent pathways for activation of capping function in parallel and can compensate for each other under some conditions. Notably, Schwer and Shuman reported that in fission yeast, an otherwise lethal mutation of Rpb1—mutation of Ser5 to Ala in all CTD repeats—can be rescued with mammalian capping enzyme, but only when capping enzyme is covalently tethered to the CTD mutant^25^. Thus, even in the absence of species-specific interactions between capping enzyme and the Pol II body, forced proximity of capping enzyme to the ternary complex, brought together by the covalent tether, is sufficient to promote efficient co-transcriptional RNA capping and, therefore, survival.

A similar model has been proposed for budding yeast, where the most stable physical interaction between capping enzyme and Pol II required two interfaces on Pol II: Ser5 phospho-CTD and the foot domain on Rpb1^22^. Disruption of either interaction caused severe growth defects in vivo and interfered with binding of capping enzyme to Pol II. A recent cryo-EM study of a ternary complex bound to the capping machinery concluded instead that capping enzyme spanned the end of the Pol II RNA exit tunnel, where it would be positioned to capture the nascent transcript as it emerges from polymerase^21^. The degree to which either of these interactions contribute directly to co-transcriptional capping in yeast or whether additional, yet-to-be-defined contacts are required remains to be determined. In any case, our observation that, when the CTD is phosphorylated, transcripts as long as 223 nt are capped as efficiently as short transcripts that have just emerged from the exit tunnel argues against a model in which optimal co-transcriptional capping requires that capping enzyme must capture the 5′-end of the nascent transcript as it emerges from Pol II.

Although we see no difference in the efficiency of capping nascent 23 and 223 nt transcripts in our purified enzyme system when the CTD is phosphorylated, capping of the longer transcripts is two–threefold less efficient when the CTD is not phosphorylated. Our findings are reminiscent of a previous report that, when the CTD is not phosphorylated, transcripts of 21 nt are capped more efficiently than longer, 31 nt transcripts in transcription complexes that had been assembled in nuclear extracts and washed with high salt^12^. However, the length dependence observed in the earlier study may be substantially greater than in our system: although the authors did not present a quantitative analysis of their capping assays, by visual inspection it appears that capping of the 31 nt transcripts required between 10- and 30-fold more enzyme to achieve similar levels of capping. There are several plausible explanations for the discrepancy, if it exists, between length dependence of capping in the two-assay systems. First, it could be a consequence of the use of different transcript sequences or lengths in our study and the earlier one. For example, our 23mers might be slightly too long for optimal capping at the proposed 21 nt sweet spot, or the 223 nt long transcripts used in our study might for some reason be more efficiently capped than 31mers. Alternatively, it could be due to the use of different Pol II transcription systems. We used a purified, reconstituted Pol II transcription system, and the earlier study used nuclear extracts; it is possible that activity(s) from the nuclear extract that either enhance capping of 21mers or inhibit capping of somewhat longer transcripts could remain associated with transcription complexes even after salt washes.

Finally, our evidence that co-transcriptional capping in artificial elongation complexes can be strongly activated by parallel pathways involving contacts with phosphorylated CTD and with the surface of Pol II may provide insight into recent findings from Nilson and colleagues^12^. They observed that capping of RNA in transcription complexes that had been assembled in nuclear extracts and washed with high salt was much less sensitive to inhibition of CTD phosphorylation with THZ1 than was capping in low salt washed complexes. Based on this observation, they proposed that the major function of CDK7 kinase in capping regulation is to promote dissociation of an activity that interferes with capping. While the identity of such an activity remains unknown, our results are consistent with the model that factor(s) bound to the body of Pol II could occlude binding site(s) for capping enzyme and thereby interfere with capping when the CTD is not phosphorylated. We believe, however, that it is not necessary to postulate that CDK7-dependent phosphorylation events are required to remove such a factor from the transcription complex. As shown by the results of our experiments using mammalian capping enzyme with *S. pombe* ternary complexes, phosphorylation of the CTD could lead to strong activation of co-transcriptional capping by compensating for the lack of contacts between capping enzyme and sites on the Pol II body rendered inaccessible by binding of the proposed factor(s) to Pol II. In the future, it will be of considerable interest to explore these issues in more detail.

## Methods

### Materials and antibodies

Unlabeled ultrapure ribonucleoside 5′-triphosphates were from GE Healthcare, 3′-O-Methyl Guanosine-5′ Triphosphate (3′-OMeGTP, cat. no. TM03-002) was from Ribomed, and [γ-^32^P] ATP, [α-^32^P] CTP, GTP, or UTP (all 3000 Ci/mmol) were from PerkinElmer. Rnasin Plus RNase Inhibitor (40 units/µl, cat. no. N2611) was from Promega. Bovine serum albumin (20 mg/ml, cat. no. B9000S), 2x RNA loading dye (cat. no. B0363S), and yeast inorganic pyrophosphatase (100 units/ml, cat. no. NEBM2403S) were from New England Biolabs. RNase T1 (1000 units/µl, cat. no. EN0541), GlycoBlue Coprecipitant (15 mg/ml, cat. no. AM9516), and Proteinase K solution (20 mg/ml, cat. no. 25530049) were from Life Technologies Invitrogen. Protease inhibitor for mammalian cell extracts (cat. no. P8340) and protease inhibitor cocktail for His-Tag purifications (cat. no. P8849) were from Sigma, and 10 mM THZ1 hydrochloride in DMSO (cat. no. HY-80013A) was obtained from MedChem Express. RNA 5′-polyphosphatase (cat. no. RP8092H), Terminator 5′-phosphate-dependent exonuclease (cat. no. TER51020), and tobacco acid pyrophosphatase (TAP; cat. no. T81050, discontinued) were from Epicentre, and decapping pyrophosphohydrolase (DppH; cat. no. 003436004, discontinued) was from Tebu-bio. Magnetic beads coupled to streptavidin (Dynabeads MyOne Streptavidin C1, Dynabeads MyOne Strepatividin T1, or Dynabeads M-280) were from Life Technologies Invitrogen. Anti-FLAG M2 affinity gel (cat. no. A2220) and FLAG peptide (cat. no. F3290) were from Sigma. MaXtract high density tubes (1.5 ml, 129046) were from Qiagen. DNA oligonucleotides were obtained from IDT (see primer table for purity specifications). 5′-triphosphorylated RNAs (containing ~15% unmodified RNA) were obtained from Trilink. Biotinylated pSer5-CTD (5×-YSPTpSPS) was synthesized at BioSynthesis (Lewisville, TX), desalted, and dissolved in 50 mM HEPES-KOH, pH 7.9, 10% DMSO. Anti-Rpb1-NTD rabbit mAb (D8L4Y; used at 1:1000 dilution) was from Cell Signaling; anti-Rpb1 antibody N-20 (sc-899; used at 1:1000 dilution) and anti-Rpb2 antibody E-12 (sc-166803; used at 1:1000 dilution) were from Santa Cruz; rat anti-RNA Pol II CTD phospho Ser5 monoclonal antibody (cat. no. 61085; used at 1:5000 dilution) was from Active Motif. IRDye 800CW goat anti-rat IgG (925–32219; used at 1:15,000 dilution) was from LiCor; donkey anti-rabbit IgG Alexa Fluor 680 (A10043; used at 1:15,000 dilution) and donkey anti-mouse IgG Alexa Fluor 680 (A10038; used at 1:15,000 dilution) were from Invitrogen.

### Preparation of RNA polymerase II and transcription factors

RNA polymerase II and TFIIH were purified from rat liver nuclear extracts^26^. Recombinant yeast TBP^27^, recombinant rat TFIIB^28^, and recombinant human TFIIE^29^ were expressed in and purified from *E. coli*^29^. TFIIF RAP30 and RAP74 subunits were amplified from human cDNA and inserted into pETDuet-1 vector (Novagen) MCS1 (His-tag) and MCS2 (no tag), respectively. Intact TFIIF was expressed in *E. coli* strain BL21(DE3)-RIL and purified on Ni-NTA agarose. Purified *S. pombe* Pol II^30^ was a gift from Henrik Spähr.

### Plasmids and immobilized templates

Plasmid pMLT-Gal4(5)-G219, made in a pGEM3 backbone, contains five 17-bp Gal4-binding sites (each separated by 2 bp) 14 bp upstream of the AdML promoter from −50 to +10, followed by a 219 bp G-less cassette. pMLT-Gal4(5)-INS20 is identical pMLT-Gal4(5)-G219 except for an insertion of “GGG” after position +20 relative to the AdML transcription start site.

The 861 bp biotinylated G23 DNA template was prepared by PCR using pMLT-Gal4(5)-INS20 as template. The 5′-primer (pMLTG5_FOR_Biotin) was biotinylated at its 5′-end and was complementary to the sequence 204 bp upstream of the first Gal4-binding site; the 3′-primer (pMLTG5_REV) was complementary to the sequence 197 bp downstream of the second G-less cassette. PCR products were purified using a QIAquick/MinElute PCR purification Kit (Qiagen).

The 99 bp biotinylated G21 DNA template contained AdML promoter sequences −36 to +10 and included a 20 bp G-less cassette. G21 template was prepared by annealing 1 nmole of non-template strand, 5′-biotinylated DNA oligo to 2 nmole of template strand DNA oligo in 50 µl of 50 mM KCl, 5 mM MgCl_2_, 25mM Tris-Cl pH 7.5. The oligo mixture was incubated for 5 min at 95 °C, then slow cooled over 72 min at 1 °C per min in a PCR machine. Biotinylated G21 DNA template was stable for at least 2 months at 4 °C.

To prepare immobilized templates, ~1 mg of M-280, MyOne C1, or MyOne T1 magnetic beads (Invitrogen) was incubated with ~15–50 pmole (10–30 µg) of biotinylated G23 template or with 1 nmole of G21 template for 30 min at room temperature in 5 mM Tris-Cl pH 7.5, 0.5 mM EDTA, 1 M NaCl, collected using a Dynamag-2 magnet (ThermoFisher), and then washed three times in the same buffer. Beads were then washed an additional three times in 20 mM HEPES-NaOH pH 7.9, 20% glycerol, 100 mM KCl, 1 mM EDTA, 0.5 mg/ml bovine serum albumin, and finally resuspended in the same buffer to a final bead concentration of 10 µg/µl. Templates immobilized on magnetic beads were kept at 4 °C and were stable for at least 6 months.

Yeast vector p-YN132 containing full-length mouse capping enzyme was a generous gift from Stewart Shuman. cDNA encoding capping enzyme was released from this plasmid by digestion with *Nde*1 and *Xho*1 and subcloned into pET15b, which encodes an in-frame N-terminal 6× HisTag.

### Expression and purification of mammalian capping enzyme

6× His-mammalian capping enzyme was expressed in *E. coli* strain BL21(DE3)-RIL and purified on Ni-NTA agarose. Capping enzyme was dialyzed for 2 h in 10K MWCO Slide-A-Lyzer cassettes (ThermoFisher) against 50 mM Tris-Cl pH 8, 50 mM NaCl, 2 mM EDTA, 10% glycerol, 5 mM sodium pyrophosphate (to deguanylate capping enzyme), and then dialyzed overnight against the same buffer lacking sodium pyrophosphate. The purity and concentration of capping enzyme was assessed on Coomassie blue-stained SDS gels, using BSA as a standard. Purified enzyme was aliquoted and kept at −80 °C.

### Promoter-dependent transcription

Unless otherwise mentioned in the figure legend, PICs were assembled for 30 min at 30 °C in 30 or 60 µl reaction mixtures containing 50–100 ng of G21 or G23 template immobilized on magnetic beads, ~10 ng of recombinant TFIIB, ~400 ng of recombinant TFIIF, ~20 ng of recombinant TFIIE, ~300 ng of TFIIH, 50 ng of yeast TBP and 0.02 units of RNA polymerase II in 3 mM HEPES-NaOH pH 7.9, 20 mM Tris-HCl pH 7.9, 60 mM KCl, 0.5 mM DTT, 0.5 mg/ml bovine serum albumin, 2% polyvinyl alcohol, 3% glycerol, 8 mM MgCl_2_ (Base Transcription Buffer or BTB), supplemented with 20 U of RNasin Plus (Promega N2611).

To synthesize 21mers, PICs assembled on G21 templates were incubated with 100 µM (Fig. 2d and Supplementary Fig. 1) or 125 µM (Figs. 1c and 2a) 3′OMeGTP, 50 µM ATP, 50 µM UTP, 2 µM CTP, 10 µCi α-^32^P-CTP (3000 Ci/mmol), 8 mM MgCl_2_, and 1 µl of T1 RNase for the indicated times.

To synthesize 23mers, 222mers, and 223mers, PICs were assembled on G23 templates immobilized on magnetic beads, and Pol II was walked to various positions by successive incubations at 30 °C with appropriate combinations of nucleotides. Transcription was initiated by addition of 25 µM ATP, 5 µM CTP, 5 µM UTP, and 10 µCi of α-^32^P-UTP (3000 Ci/mmol). After 5 min, reactions were supplemented with an additional 50 µM ATP, 50 µM CTP, and 50 µM UTP and incubated an additional 2 min to generate 20mers. Immobilized transcription complexes were collected and washed twice with a volume of high salt wash buffer (HSB, containing 3 mM HEPES-NaOH pH 7.9, 20 mM Tris-HCl pH 7.9, 1 M KCl, 0.5 mM DTT, 0.5 mg/ml bovine serum albumin, 0.2% polyvinyl alcohol, and 3% glycerol) equivalent to the initial reaction volume, followed by two more washes in low salt wash buffer (LSB, containing 3 mM HEPES-NaOH pH 7.9, 20 mM Tris-HCl pH 7.9, 60 mM KCl, 0.5 mM DTT, 0.5 mg/ml bovine serum albumin, 0.2% polyvinyl alcohol, and 3% glycerol). Transcription complexes were then incubated for 2 min in BTB containing 5 µM GTP to generate 23mers, washed twice with HSB and twice with LSB. When necessary, transcription complexes containing 23mers were resuspended in BTB containing 500 µM ATP, 500 µM CTP, and 500 µM UTP, incubated for 30 min to allow synthesis of 222mers and washed as described above. Washed transcription complexes containing 23mers or 222mers were used as substrates for RNA capping reactions or analyzed by denaturing polyacrylamide gel electrophoresis. 222 nt transcripts were extended to 223mers during capping reactions, since GTP used as capping substrate allowed for addition of one nucleotide to transcripts.

Where indicated, 150 µM THZ1 was included during PIC assembly and synthesis of 20mers or 21mers; to control for solvent effects an equivalent volume of DMSO was included in control reactions for these experiments. In the experiments of Figs. 2b, c and 7, all subsequent steps, except for washes, included 50 µM THZ1, even in control reactions, to control for off-target effect(s) of THZ1.

### Artificial Pol II elongation complexes

For assembly of artificial elongation complexes, 1 nmole of non-template DNA was immobilized on magnetic beads and washed as described above. Immobilized oligo was stable for at least 6 months at 4 °C. To begin assembly of artificial elongation complexes, 20 pmol of RNA 20mer oligo with a 5′-triphosphate (RNA_20mer) were annealed to 10 pmol of template strand DNA oligo (TS_DNA) in 10 µl 25 mM Tris pH 7.5, 50 mM KCl, and 5 mM MgCl_2_. Reactions were incubated 5 min at 45 °C, then incubated for 12 cycles of 2 min each, starting at 43 °C and decreasing the temperature 2 °C per cycle in a PCR machine. All further incubations were at 30 °C. One pmol of template strand:RNA hybrid was incubated with 0.02 units of Pol II in 50 mM Tris pH 7.5, 50 mM KCl, 5 mM MgCl_2_, 2% polyvinyl alcohol, 3% glycerol, 0.5 mg/ml BSA, and 0.5 mM DTT for 10 min. An equal volume of the same buffer supplemented with 5 pmol of non-template strand DNA oligo (NTS_DNA) immobilized on magnetic beads was then added to the reaction and incubated for 10 min at 37 °C. Immobilized transcription bubbles were then washed by collecting samples in magnet for 2 min, then resuspended in an equal volume of LSB. To generate transcription complexes containing radiolabeled 23mers, immobilized transcription bubbles were collected, resuspended in 25 µl of BTB containing 0.6 µM ATP and 10 µCi of α-^32^P-UTP (3000 Ci/mmol), and incubated for 10 min. Following this incubation, 5 µl of BTB supplemented with 5 µM ATP and 5 µM UTP was added, reactions were incubated a further 5 min, and the resulting transcription complexes were washed twice with 3 mM HEPES-NaOH pH 7.9, 20 mM Tris-HCl pH 7.9, 60 mM KCl, 0.5 mM DTT, 0.5 mg/ml bovine serum albumin, 0.2% polyvinyl alcohol, and 3% glycerol.

Washed transcription complexes were processed differently depending on the nature of the experiment. To walk Pol II along the template to generate transcripts of the desired lengths, transcription complexes were resuspended in BTB supplemented with the appropriate combinations of 20 µM NTPs and incubated for 10 min. For phosphorylation by TFIIH, transcription complexes were resuspended in BTB supplemented with 50 µm ATP and 1 µl (~300 ng) of purified TFIIH for 10 min. For capping, washed complexes were processed as described below. In multistep reactions, such as that of Fig. 3b, transcription complexes were washed twice with 3 mM HEPES-NaOH pH 7.9, 20 mM Tris-HCl pH 7.9, 60 mM KCl, 0.5 mM DTT, 0.5 mg/ml bovine serum albumin, 0.2% polyvinyl alcohol, and 3% glycerol between steps.

### Free RNA and RNA capping

Radiolabeled 23mer RNA was generated using artificial Pol II elongation complexes and purified by phenol–chloroform–isoamyl alcohol extraction, chloroform extraction, and ethanol precipitation as described below. Purified RNA was resuspended in 5 µl of H_2_O per reaction and used as a substrate for capping reactions.

Free RNA or washed transcription complexes with transcripts initiated from the promoter on the G23 template or transcripts in artificial ternary complexes were suspended in BTB supplemented with 50 μM GTP and 0.1 unit of inorganic yeast polyphosphatase and then transferred to new tubes containing the indicated amounts of capping enzyme. To assay capping of transcripts initiated from the promoter on the G21 template, capping enzyme was added to pre-assembled PICs along with nucleotides for RNA synthesis; note that these reactions included 100–125 µM 3′OMeGTP instead of GTP.

### RNA purification and analysis

Transcription or capping reactions were stopped by addition of 60 μl 10 mM Tris-HCl (pH 7.5), 300 mM NaCl, 0.5 mM EDTA, 0.2% SDS and 2 µl of 20 mg/ml proteinase K, 2 µl of GlycoBlue 15 mg/ml (Invitrogen AM9516), and enough H_2_O the final volume of solution to 124 µl, including the volume of the original reaction mix. Samples were then extracted once with 124 µl of phenol:choloroform:isoamyl alcohol (25:24:1) and once with chloroform:isoamyl alcohol (24:1) using MaXtract high density tubes (Qiagen), brought to 0.3 M sodium acetate by addition of 12.4 µl of 3 M sodium acetate pH 5.2, ethanol precipitated, and washed with 70% ethanol. After removal of the final ethanol wash, RNA pellets were air dried for 3 min, resuspended in 1× RNA loading dye, heated at 70 °C for 10 min, spun 4 min at 2000 × *g*, and resolved on a denaturing gel (15% PAGE 1:19 bis/tris, 7.0 M urea). Radiolabeled gels were exposed to a phosphorimager (Molecular Dynamics or Amersham Biosciences) and scanned using a Typhoon Trio imager (Amersham Biosciences). Images were quantified using ImageQuantTL (GE Healthcare) and plotted using Graphpad Prism (version 6.05).

The percent of capped RNA was determined by measuring the ratio of capped RNA signal/total RNA (capped + uncapped) and normalized to the maximum obtainable capping. Maximum capping of transcripts initiated from promoter was consistently 100%. For analysis of free RNA or artificial Pol II elongation complexes, the maximum % capped RNA was determined to be 85%, likely due to incomplete triphosphorylation of synthetic RNA.

### Immunoblotting

Protein samples were boiled for 10 min at 70 °C, and loaded into 5% handcast SDS-PAGE gels or commercially available pre-cast gels (Bio-Rad 3450002). After electrophoresis, proteins were transferred to a PVDF membrane (Millipore Immobilon-FL). Membranes were blocked for 30 min using Odyssey Blocking Buffer PBS (LICOR), incubated overnight with primary antibody that had been diluted in Odyssey Blocking Buffer supplemented with 0.1% Tween-20, washed with TBS containing 0.05% Tween-20, incubated with the appropriate secondary antibodies at room temperature, washed again, and then finally scanned using a LICOR Odyssey Scanning Instrument. Supplementary figure 7 includes uncropped images of the most important blots and gels.

### Purification of human Pol II containing RPB1 lacking the CTD

Gibson assembly was used to generate a DNA fragment encoding RPB1 lacking the CTD (RPB1-ΔCTD, amino acids 1–1592) from three separate DNA fragments. Fragment 1 included a 5′ *Xho*I site followed by nucleotides 1–1449 of NM_000937 CDS; fragment 2 included nucleotides 1425–2829, and fragment 3 included nucleotides 2808–4776 followed by stop codon and *Bam*HI site; these were synthesized by PCR, assembled, and cloned into pcDNA5 (Life Technologies). This insert was subcloned into a modified pcDNA5/FRT/TO vector that encodes an in-frame N-terminal 3×FLAG tag, and co-transfected with pOG44 into Flip-In T-Rex 293 cells with FuGENE6 (Promega). Stably transfected cells were selected using 100 µg/ml of hygromycin. Hygromycin-resistant cell clones were treated with 2 µg/ml of doxycycline for 48 h to induce RPB1-ΔCTD, and protein expression was confirmed by immunoblot with anti-FLAG mAb.

Intact nuclei were prepared essentially as described^31^. Briefly, stable Flip-In T-Rex 293 F:RPB1-ΔCTD cells were grown to near confluence in four roller bottles, treated with 2 µg/ml doxycycline, and harvested 24 h later. Cells were lysed with 10 mM HEPES-NaOH pH 7.9, 0.34 M sucrose, 3 mM CaCl_2_, 2 mM magnesium acetate, 0.1 mM EDTA, 1 mM DTT, 0.5% Nonidet P-40, and protease inhibitors (Sigma), strained through a 70-µM filter, and pelleted by centrifugation. Nuclei were washed with the same buffer without detergent before continuing with preparation of total nuclear extracts^32^. Nuclear extracts were applied to M2-agarose beads, and F:RPB1–ΔCTD protein complex was purified as previously described^33^.

### Assay for formation of GMP-capping enzyme intermediate

Free RNA or washed artificial ternary complexes were suspended in BTB supplemented with 0.1 unit of inorganic yeast polyphosphatase and 10 µCi of α-^32^P-GTP (3000 Ci/mmol) instead of GTP, and then transferred to new tubes containing 5 ng of capping enzyme. Reactions were stopped with 1× Laemmli Sample Buffer at the indicated times, boiled for 10 min at 70 °C, and loaded into 4–20% gradient gel (Bio-Rad). After electrophoresis, gels were fixed in 40% methanol, 10% acetic acid for 1 h, and rinsed with water. Radiolabeled gels were exposed to a phosphorimager and processed as described above.

In the experiment of Fig. 6e, f, washed artificial ternary complexes containing 23mers were resuspended in 5 mM DTT, 50 mM Tris-Cl pH 7.9, 5 mM MgCl_2_, 50 µM GTP, 0.1 unit of inorganic yeast polyphosphatase, and 10 µCi of α-^32^P-GTP (3000 Ci/mmol) or 0.22 µM GTP for GTase and capping assay, respectively, and then transferred to new tubes containing 15 ng of capping enzyme and increasing amounts of pSer5-CTD peptide. Reactions were incubated for 4 min at 37 °C then stopped with 1x Laemmli Sample Buffer and processed as above.

### Kinase assay

Washed transcription complexes with transcripts initiated from the promoter on the G23 template (Supplementary Fig. 2A) or transcripts in artificial ternary complexes (Supplementary Fig. 4A) were suspended in BTB supplemented with 100 µM 3′OMeGTP, 50 μM ATP, 50 µM CTP, 2 µM UTP, and 10 µCi of γ-^32^P-ATP. Reactions were incubated for 15 and 60 min, washed twice with HSB then twice with LSB (for Supplementary Fig. 2A) or just twice with LSB (for Supplementary Fig. 4A) before stopping with 1× Laemmli Sample Buffer. Samples were then boiled for 10 min at 70 °C, and loaded onto 4–15% gradient gels (Bio-Rad). After electrophoresis, gels were fixed in 40% methanol, 10% acetic acid for 1 h, and rinsed with water. Radiolabeled gels were exposed to a phosphorimager and processed as described above.

### Data availability

All original data underlying this paper can be accessed from the Stowers Original Data Repository at http://www.stowers.org/research/publications/LIBP-1278.

## Electronic supplementary material

Supplementary Information
