# Supplementary material for: CTD-dependent and -independent mechanisms govern co-transcriptional capping of Pol II transcripts
Source: Nat Commun. 2018 Aug 23;9:3392. doi: 10.1038/s41467-018-05923-w (PMC6107522; doi:10.1038/s41467-018-05923-w)
Supplement: Supplementary file 1 — Supplementary Information [file 41467_2018_5923_MOESM1_ESM.pdf]

Supplementary Information for

CTD-dependent and -independent mechanisms govern co-transcriptional capping of Pol II transcripts

by Noe Gonzalez, et al.

Table 1. Primers and Oligos

TFIIF-1_F_NdeI-NoTag	AGTCCATATGGCGGCCCTAG[1]	For RAP74 cloning
TFIIF-1_R_Kpn1-NoTag	ACTAGGTACCTCACTCCTTGAGGGA[1]	For RAP74 cloning
TFIIF-2_F_EcoRI-HisTag	GTACGAATTCAATGGCCGAGCG[1]	For His-RAP30 cloning
TFIIF-2_R_HindII-HisTag	CAGAAGCTTAGTCACTCTTTTCTTCTCCTT[1]	For His-RAP30 cloning
pMLTG5_FOR_Biotin	TAGAGGATCTGGCTAGCG	5' TEG-biotinylated, HPLC purified
pMLTG5_REV	TGTGGATAACCGTATTACCG	HPLC purified
NonTemplate_Control_G20	TCGGTACCGGTGTTCCTGAAGGGGGCTATAAAAGGGGGTGGGGGCGCGTTCGTCCTCACTCTCTTCCTCTCTTCTTAGACTCAGGCATGCAAGCTTGGC	5' Biotinylated, HPLC purified
TemplateStrand_G20	GCCAAGCTTGCATGCCTGAGTCTAAGAAGAGAGGAAGAGAGTGAGGACGAACGCGCCCCCACCCCCTTTTATAGCCCCCTTCAGGAACACCGGTACCGA	PAGE purified.
RNA_20mer	ACUCUCAUCUCUCAUCCUUA	5' Triphosphate modification, PAGE & RP-HPLC purified
TS_DNA	CTACGGTTAAGCTCACGGTACATTTCTGAATTAAGGATGATGG	Dual PAGE & HPLC purified.
NTS_DNA	ATCAGAAATGTACCGTGAGCTTAACCGTAG	5' TEG-biotinylated, HPLC purified

[1] Underlined sequences include restriction sites used for cloning.

Supplementary Fig. 1, Noe Gonzalez et al 2018

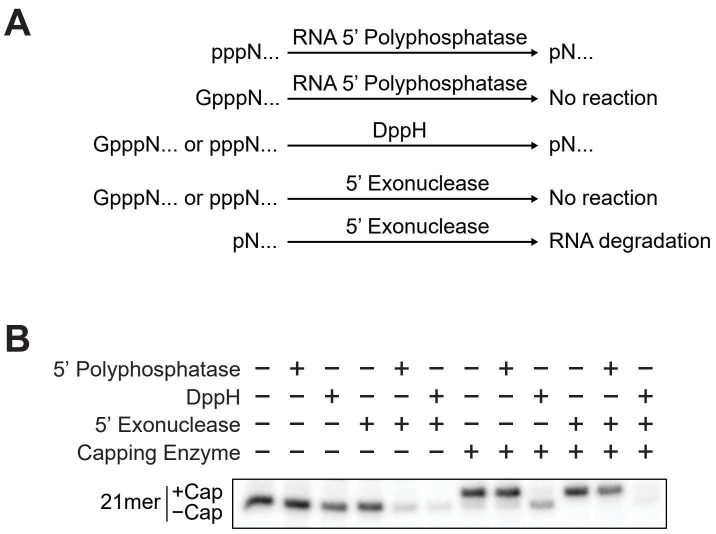

Supplementary Figure 1. Enzymatic cleavage of capped RNA. ***A,*** Diagrams show products of reactions catalyzed by each enzyme in the presence of capped (GpppN...) or uncapped (pppN...) RNAs. RNA 5' polyphosphatase converts 5' triphosphate (pppN) to 5' monophosphate (pN) only when RNA is uncapped; Decapping pyrophosphohydrolase (Dpph) converts 5' triphosphate to 5' monophosphate when RNA 5' ends are capped or uncapped; 5' exonuclease degrades only RNA having 5' monophosphate ends. ***B,*** Synthesis and capping of 21mers associated with transcription complexes initiated from the AdML were performed in reactions containing 100 µM 3'OMeG and 100 ng of capping enzyme for 30 min, and RNA was purified as described in Materials and Methods. Dephosphorylation reactions were carried out for 1 hr at 30 °C in 20 µl reaction mixtures containing purified radiolabeled RNA, 2 µl of the appropriate 10 x enzyme buffer provided with each enzyme, with or without 2 µl of 5' RNA polyphosphatase (Epicentre) or 2 µl of DppH (Tebu-bio). RNA was purified again and incubated for 1 hr at 30 °C in 20 µl reactions containing 2 µl 10x exonuclease buffer, with or without 2 µl of 5' exonuclease.

Supplementary Fig. 2, Noe Gonzalez et al 2018

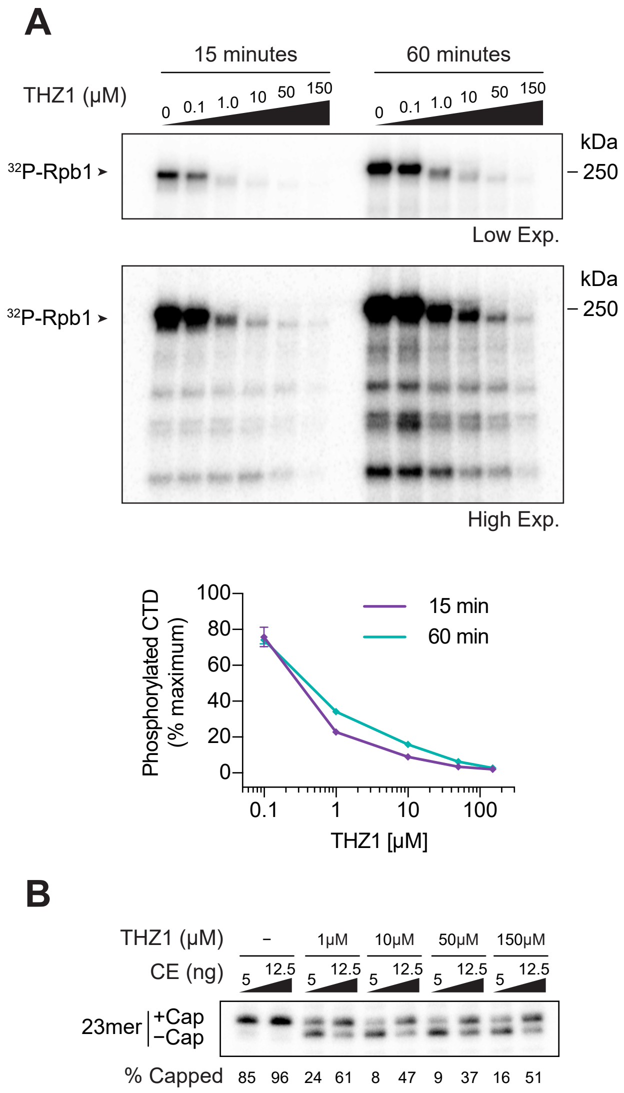

Supplementary Figure 2. THZ1 inhibits CTD phosphorylation and capping during promoter-specific transcription. ***A,*** 21mers initiated from the AdML promoter (G23) were synthesized in reactions containing unlabeled ribonucleoside triphosphates supplemented with 10 µCi γ-^{32}P ATP in the presence of DMSO (-) or increasing amounts of THZ1; reactions were washed and stopped after 15 or 60 min. Upper panel, reaction products were separated on SDS-PAGE gels and visualized by phosphorimaging; two different exposures of the same image are shown. The graph shows the mean and range of two independent reactions. ***B,*** Washed transcription complexes containing 23mers synthesized with or without the indicated concentrations of THZ1 were incubated for 4 min with capping enzyme (CE) and GTP. % Capped indicates average of two independent reactions.

Supplementary Fig. 3, Noe Gonzalez et al 2018

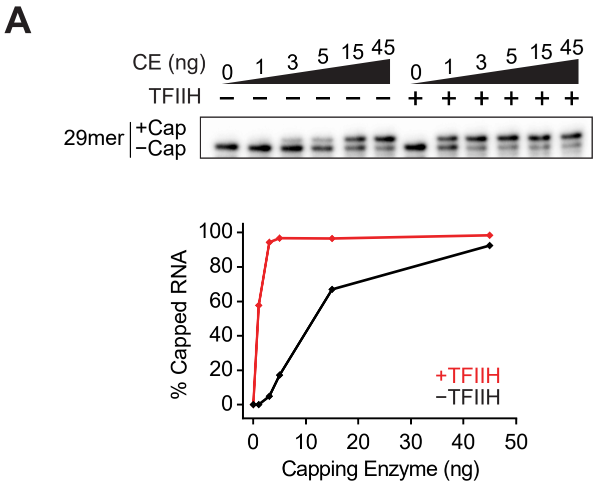

Supplementary Figure 3. Titration of capping enzyme shows TFIIH-dependent activation of capping in artificial ternary complexes. ***A,*** Artificial ternary complexes containing 29mers were incubated with or without 300 ng of purified TFIIH for 10 minutes, washed, and resuspended in buffer with 50 µM GTP and the indicated amounts of capping enzyme. Capping reactions were stopped after 15 min. The graph shows quantification of RNA capping in these reactions.

Supplementary Fig. 4, Noe Gonzalez et al 2018

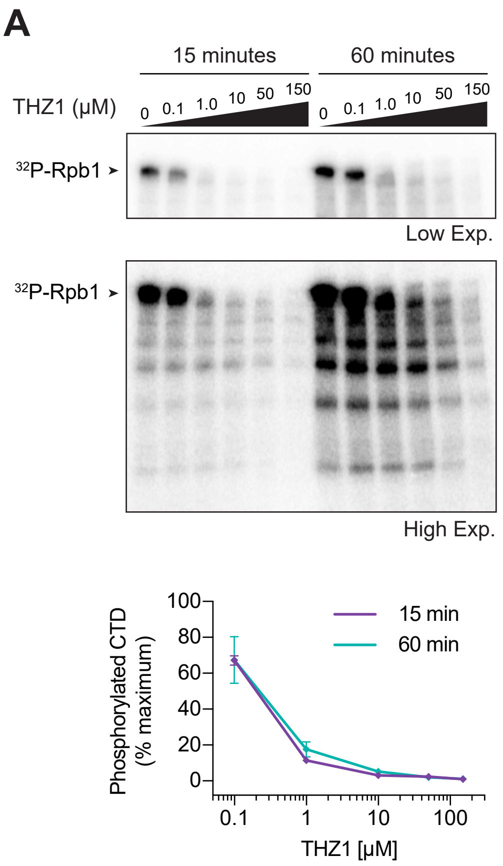

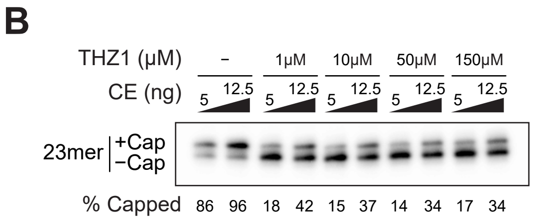

Supplementary Figure 4. THZ1 inhibits CTD phosphorylation and capping in artificial ternary complexes. ***A,*** Artificial ternary elongation complexes with 23mers were pre-incubated with 300 ng of purified TFIIH and DMSO (-) or the indicated amounts of THZ1 for 30 min and then supplemented with unlabeled ribonucleoside triphosphates and 10 μCi γ-^{32}P ATP; reactions were washed and stopped after 15 or 60 min. Upper panel, reaction products were separated on SDS-PAGE gels and visualized by phosphorimaging; two different exposures of the same image are shown. The graph shows the mean and range of two independent reactions. ***B,*** Washed artificial ternary complexes containing 23mers were pre-incubated with 300 ng of purified TFIIH and DMSO (-) or the indicated amounts of THZ1 for 30 minutes, and then supplemented with ATP for 10 mins. Ternary complexes were washed and incubated with the same concentrations of THZ1 or DMSO, GTP, and the indicated amounts of capping enzyme (CE) for 4 min before stopping. % Capped indicates average of two independent reactions.

Supplementary Fig. 5, Noe Gonzalez et al 2018

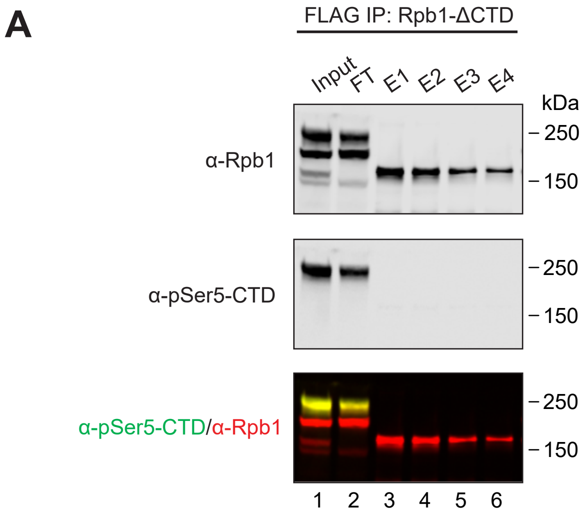

Supplementary Figure 5. Purification of Pol II lacking the CTD from stable cell lines. ***A,*** Western blot showing endogenous Rpb1 and F:Rpb1-ΔCTD through the stages of flag immunopurification using antibodies against total Rpb1 (α-Rpb1) or Ser5 phosphorylated Rpb1 CTD (α-pSer5-CTD). See "Materials & Methods" for details. FT, FLAG agarose flow through fraction; E1 through E4, fractions obtained during elution with FLAG peptide from anti-FLAG agarose.

Supplementary Fig. 6, Noe Gonzalez et al 2018

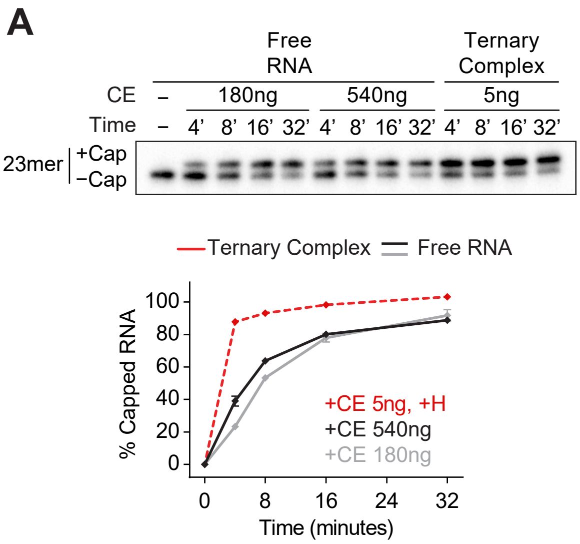

Supplementary Figure 6. Comparison of capping kinetics between ternary complexes and free RNA. Capping kinetics comparison between ***A*,** Kinetics of capping of free 23mer RNA or 23mers in phosphorylated artificial ternary complexes. Reactions contained 50 µM GTP and the indicated amounts of capping enzyme. The first 5 lanes are the same as those used in Fig 1C, Free RNA. Graph shows values for capping in ternary complexes from the single experiment shown in the figure and the mean and range from 2 independent experiments for free RNA capping reactions.

Supplementary Fig. 7, Noe Gonzalez et al 2018

A

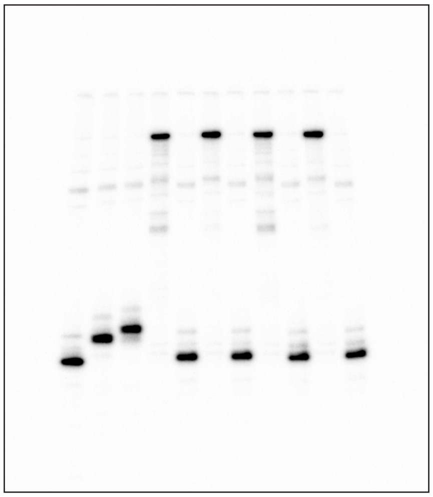

B

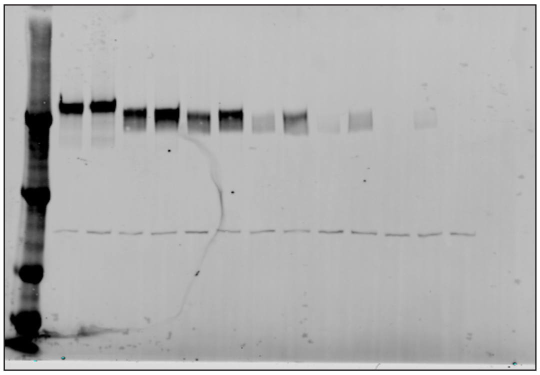

C

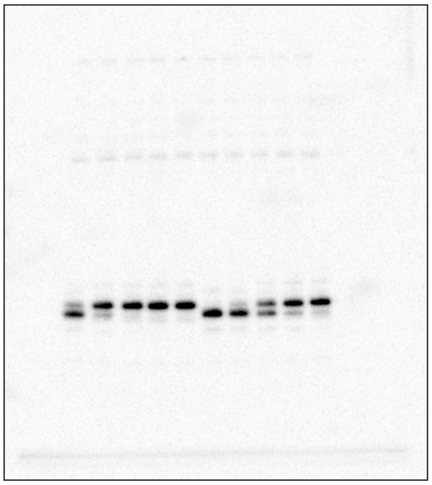

D

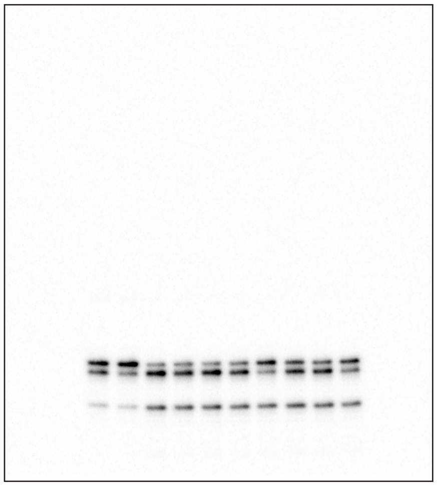

E

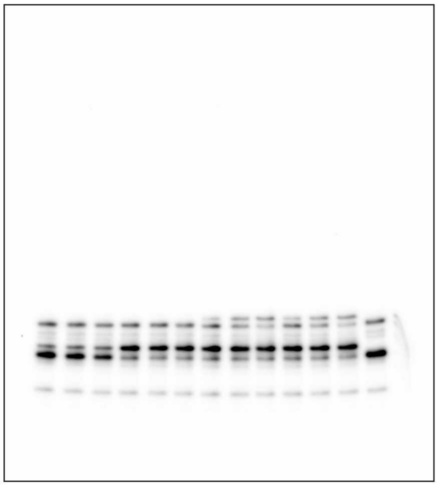

F

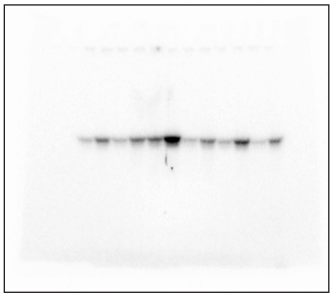

G

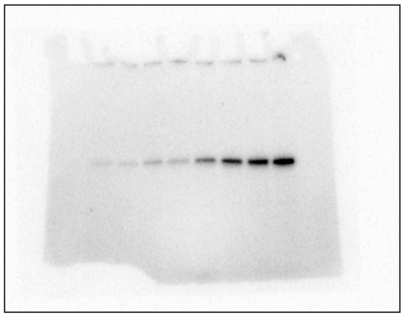

H

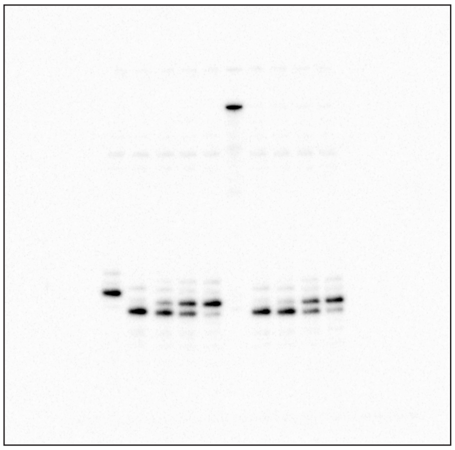

Supplementary Figure 7. Uncropped gels and blots.
A, Uncropped Figure 1B and 7A
B, Uncropped Figure 2A (Western Blot).
C, Uncropped Figure 2B.
D, Uncropped Figure 4B.
E, Uncropped Figure 6A.
F, Uncropped Figure 6C (GTase Assay).
G, Uncropped Figure 6E (GTase Assay).
H, Uncropped Figure 7C.
